# The ecological niche of reported rabies cases in Canada is similar to Alaska

**DOI:** 10.1111/zph.12835

**Published:** 2021-05-06

**Authors:** Falk Huettmann, Karsten Hueffer

**Affiliations:** ^1^ EWHALE lab‐ Inst of Arctic Biology Department of Biology & Wildlife University of Alaska Fairbanks AK USA; ^2^ Department of Veterinary Medicine University of Alaska Fairbanks AK USA

**Keywords:** Alaska, Canada, geographic information system, Machine Learning, Rabies

## Abstract

The ecology of rabies in the circumpolar North is still not well understood. We use machine learning, a geographic information system and data explicit in time and space obtained for reported rabies cases and predictors in Canada to develop an ecological niche model for the distribution of reported rabies cases in the American north (Alaska and Canada). The ecological niche model based on reported rabies cases in Canada predicted reported rabies cases in Alaska, suggesting a rather robust inference and even similar drivers on a continental scale. As found in Alaska, proximity to human infrastructure—specifically along the coast—was a strong predictor in the detection of rabies cases in Canada. Also, this finding highlights the need for a more systematic landscape sampling for rabies infection model predictions to better understand and tackle the ecology of this important zoonotic disease on a landscape scale at some distance from human infrastructure in wilderness areas.

**Impacts**

- First ecological niche model of reported rabies cases in Canada using currently available data obtained primarily from public health surveillance efforts and public geographic information system layers.
- Valuable model built with reported rabies cases in Canada predicts reported rabies cases in Alaska suggesting a similar distribution of reported rabies cases for these two geographic regions on a continental scale, especially in the circumpolar regions.
- Human infrastructure is associated with reported rabies cases, specifically in coastal regions. However, to fully understand rabies ecology independent of possible human exposures in Canada and Alaska a more systematic sampling regime is suggested for further confirmation.

## 1 INTRODUCTION

Rabies is a disease of public health concern in many regions including the circumpolar North (Hueffer & Murphy, 2018; Hueffer et al., 2013). In North America, rabies is maintained in wildlife populations which serve as reservoirs of different rabies virus variants (RVV) (Ma et al., 2018). Rabies is enzootic in wildlife and the maintenance hosts in North America are bats, foxes, skunks and raccoons, although the distribution of RVVs differs geographically (Fehlner‐Gardiner, 2018). Bat RVVs are enzootic throughout southern Canada, with distinct variants circulating in different bat species (Nadin‐davis et al., 2001, 2010, 2017). The RVVs that are enzootic in the Arctic and possibly red fox populations in the Canadian north belong to the Arctic lineage of rabies viruses (Nadin‐Davis et al., 2008). Currently, Arctic‐3 (A3) RVV is the main virus circulating in the Canadian North. The Arctic‐1 (A1) RVV, which previously spread to and circulated in red foxes and skunks in southern Ontario, was presumed eliminated in 2012, as no cases were detected for several years, but detections since late 2015 have shown this not to be the case (Government of Ontario, 2019; Nadin‐Davis & Fehlner Gardiner, 2019). In the Canadian prairie provinces, the enzootic terrestrial RVV is associated with the striped skunk (Davis et al., 2013), whereas in the provinces of Ontario, Quebec and New Brunswick, epizootics caused by the raccoon RVV have been detected in some areas bordering the United States since 1999 (Stevenson, Goltz, & Massé, 2016).

Diagnostic testing is carried out in centralized, federal laboratories of the Canadian Food Inspection Agency (CFIA) when a rabies‐suspected animal has exposed a person or a domestic animal. Some provinces carry out enhanced surveillance and wildlife rabies control programs. Over the last 15 years, CFIA diagnosed an average of 202 rabies cases in animals per year (range 92–392), whereas in the 1980s, prior to implementation of fox rabies control programs in Ontario, approximately 2,000 cases/year were reported (Rosatte, 1988).

The detection of rabies in Alaska shows a distinct distribution of cases, with areas where rabies is considered endemic and areas where occasional outbreaks have occurred (Huettmann et al., 2017). In northern and western Alaska, rabies is maintained by foxes, while in southeast Alaska bats are the main reservoir hosts (Hueffer & Murphy, 2018). Three distinct RVVs of the Arctic virus lineage are maintained in Alaska (Arctic‐2, Arctic‐3 and Arctic‐4) (Goldsmith et al., 2016; Kuzmin et al., 2008). Population genetic analyses suggest that the arctic fox is the main reservoir of rabies in Alaska, possibly in a multi‐host system together with the red fox (Goldsmith et al., 2016). Several lines of evidence suggest that human exposure to arctic RVVs under the current ecological conditions could decline under climate change scenarios, although uncertainty remains around these predictions of future distribution of rabies in Alaska (Hueffer & Murphy, 2018; Huettmann et al., 2017; Kim et al., 2014).

Previous studies aimed at the use of ecological niche modelling techniques to elucidate reported terrestrial rabies case distribution in Alaska between 1914 and 2013 identified variables such as proximity to human infrastructure and climatic variables that well‐predicted reported rabies cases (Huettmann et al., 2017). Rabies case detection in both Alaska and Canada is mainly focused on a public health‐based, passive surveillance model. Rabies is sampled opportunistically with public health responses, rather than research design, as the main driver of sampling (Huettmann et al., 2017). However, while biased towards proximity to humans, such data can form the basis for useful models (Drew et al., 2011; Huettmann et al., 2017; Humphries et al., 2018; Kadmon et al., 2004). In this study, we describe the ecological niche of reported rabies cases based on the best currently available data from Canada explicit in space and time, and we investigate the dynamics of reported rabies cases in Canada compare to those in Alaska. We point out the need for more unbiased sampling to better understand the ecology of rabies independent of human exposure.

## 2 METHODS

We obtained 1,788 georeferenced reported rabies cases from 2007 to 2016, from the CFIA databases. The majority of the cases are in animals that had exposed either a person or a domestic animal. The data also include animals that were collected during enhanced surveillance programmes carried out by certain provinces in eastern Canada. Geographic information system (GIS) location data were validated, and a small number of cases that had location co‐ordinates that either fell outside of Canada or not on land were corrected based on the town name provided in the data set for these cases.

We used ArcGIS 10.3 and QGIS to map the rabies cases in a WGS84 geographic projection with decimal latitude and longitude to six decimals as a default setting. We then overlaid this data set with 11 predictors (elevation, precipitation in January, precipitation in July, mean temperature in January, mean temperature in July, human population density, mammal diversity, proximity to coast, proximity to roads, proximity to rivers, human footprint index; for data see Sriram and Huettmann unpublished https://www.earth‐syst‐sci‐data‐discuss.net/essd‐2016‐65/).

We used the default settings in Salford Predictive Modeling Suite (SPM8; https://www.salford‐systems.com/ Random Forest, Treenet) to data mine and predict the data cube (Drew et al., 2011; Huettmann et al., 2017; Humphries et al., 2018; Ohse et al., 2009). We employed that niche in SPM as a grove file to a regular lattice of 1km spacing for all of North America, which we then smoothed with an Inverse Distance Weighting (IDW) algorithm in GIS to obtain a smoothed out gridded surface from the point lattice. We overlaid this predicted surface to Alaska and assessed how well the Alaska data performed on this independent model trained by Canadian data. We extracted the pixel values for previously reported cases in Alaska (Huettmann et al., 2017) and computed the matching per cent between both data sets.

Details on modelling are provided online at https://scholarworks.alaska.edu/handle/11122/11015.

## 3 RESULTS

**FIGURE 1:**
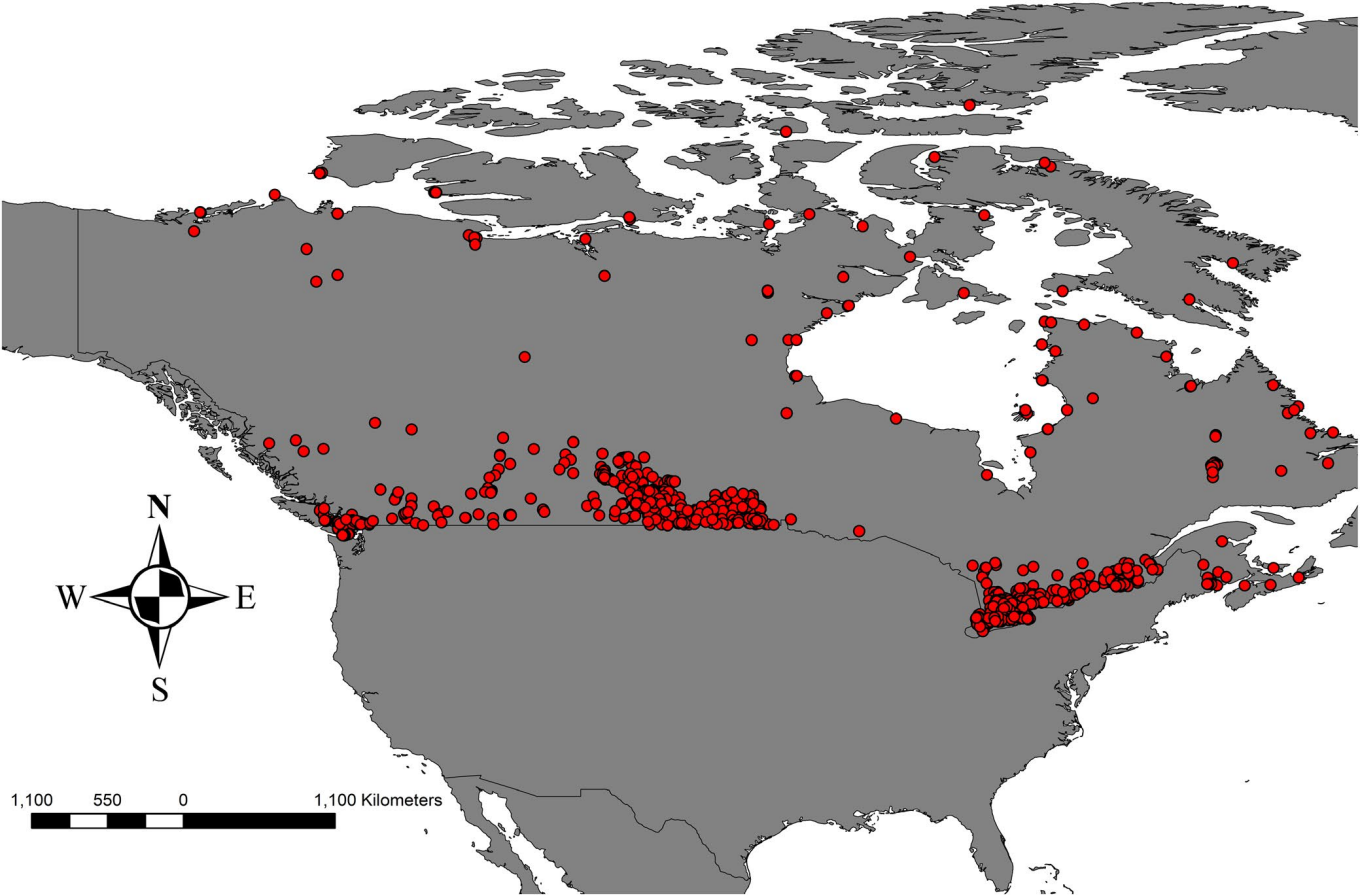
Distribution of reported rabies cases in Canada 2007–2016 included in subsequent data mining and machine learning modelling

We curated 1,788 reported cases from CFIA 2007–2016 in different host species (Figure 1, Data S1).

Rabies cases in Canada were distributed primarily among four regions within the country: (a) North, that is, Northwest Territories, Nunavut, northern Manitoba, northern Ontario, northern Quebec (Nunavik) and Labrador; (b) West, that is, British Columbia and Alberta; (c) Prairie, that is, Saskatchewan and southern Manitoba; and (d) East, that is, southern Ontario, southern Quebec and the Atlantic provinces. The proportion of rabies cases in each region, by species, is shown in Table 1. The majority of rabies cases (~ 90%) in Canada were recorded in wildlife species, with bats (33.1%) and skunks (30.1%) being the species most commonly found rabid. Spatially though, the species of importance vary, with rabid foxes being most significant the northern region. In all regions, cross‐species transmission (spill‐over) cases were detected in domestic animals (livestock and/or pets) or non‐reservoir wildlife species.

**TABLE 1:** Per cent of rabies cases by species, for each region of Canada, 2007–2016

Species	Region				Country
	North	West	Prairie	East	
Arctic fox	47.2	—	—	—	5.7
Red fox	27.8	—	—	1.0	3.8
Raccoon	—	—	0.4	32.1	16.3
Skunk	—	—	68.7	19.5	30.6
Wolf	5.6	—	—	—	0.7
Bats	—	98.5	14.5	42.5	33.1
Livestock^a^	—	—	7.6	3.7	4.2
Pets^b^	19.4	1.5	8.8	1.2	5.7

^a^ Cows, donkeys, goats, horses, llamas, sheep.

^b^ Dogs and cats.

**FIGURE 2:**
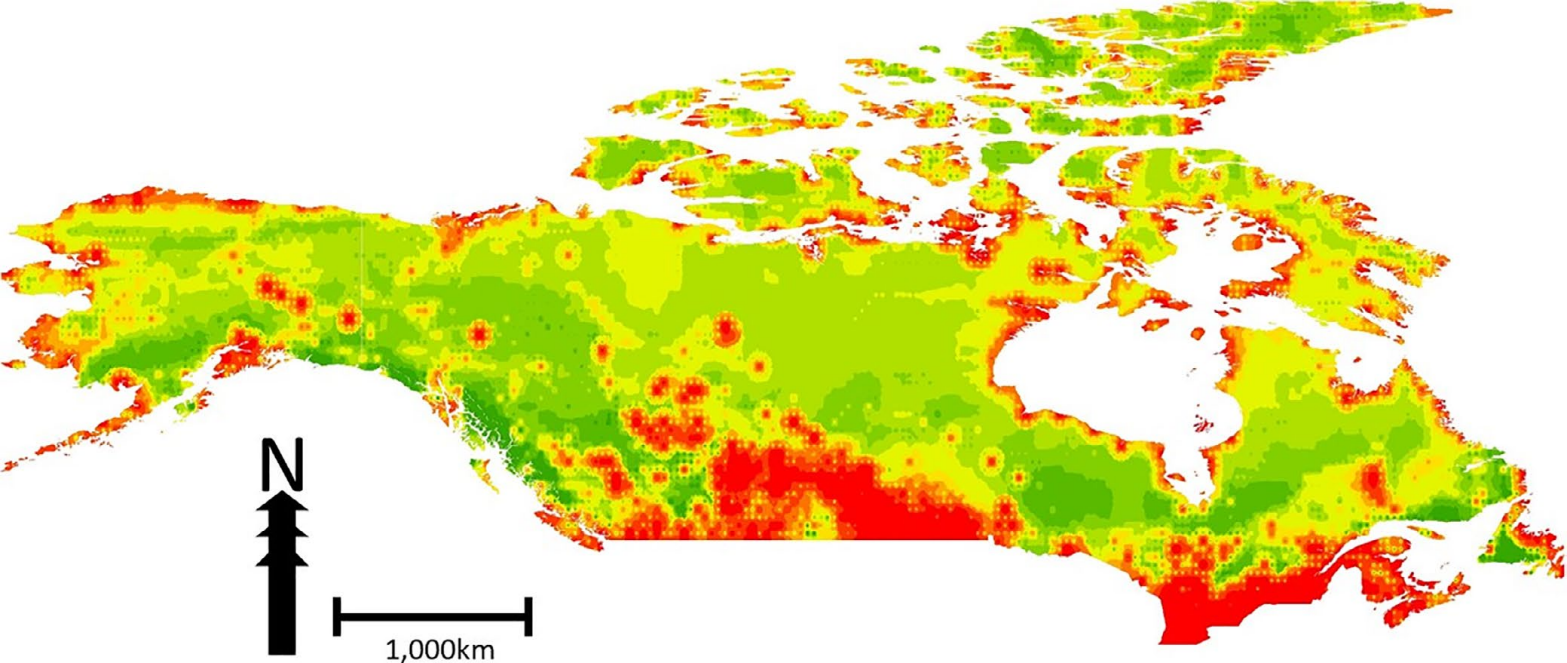
Predicted relative index of occurrence (RIO) of reported rabies for Canada and Alaska based on reported rabies cases in Canada. Red indicates high RIO, green indicates low RIO

Using these data, 11 GIS predictor layers, and machine learning algorithms we predicted the relative occurrence of rabies in Canada (Figure 2). The accuracy, based on Receiver Operating Characteristics (ROC) analysis (Humphries et al., 2018), of this model is approximately 98% (model‐based) with a pixel resolution of *c*. 3.7 km.

**FIGURE 3:**
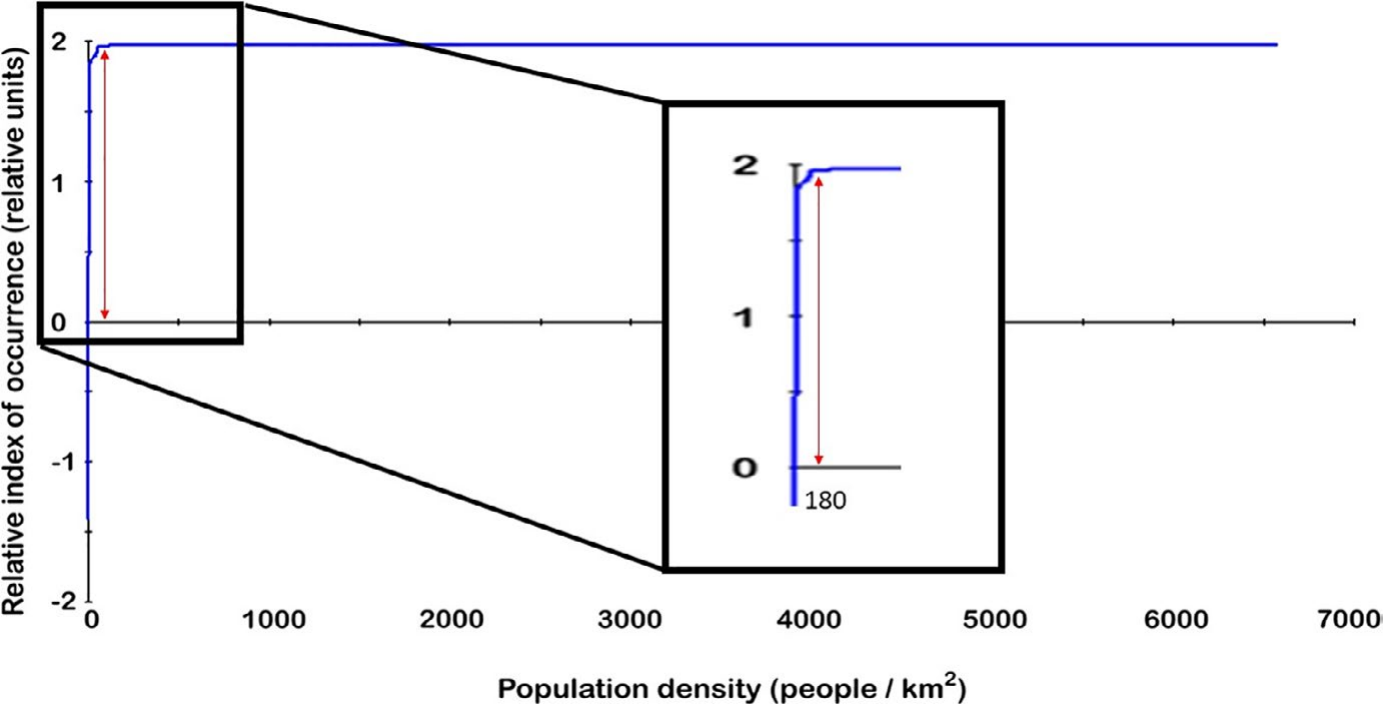
Relative index of occurrence (RIO) of reported rabies cases as a function of human population density in the model output from model based on reported rabies cases in Canada. RIO is related to human populations although heavily biased sampling for possible human exposure has to be considered when interpreting this association

**FIGURE 4:**
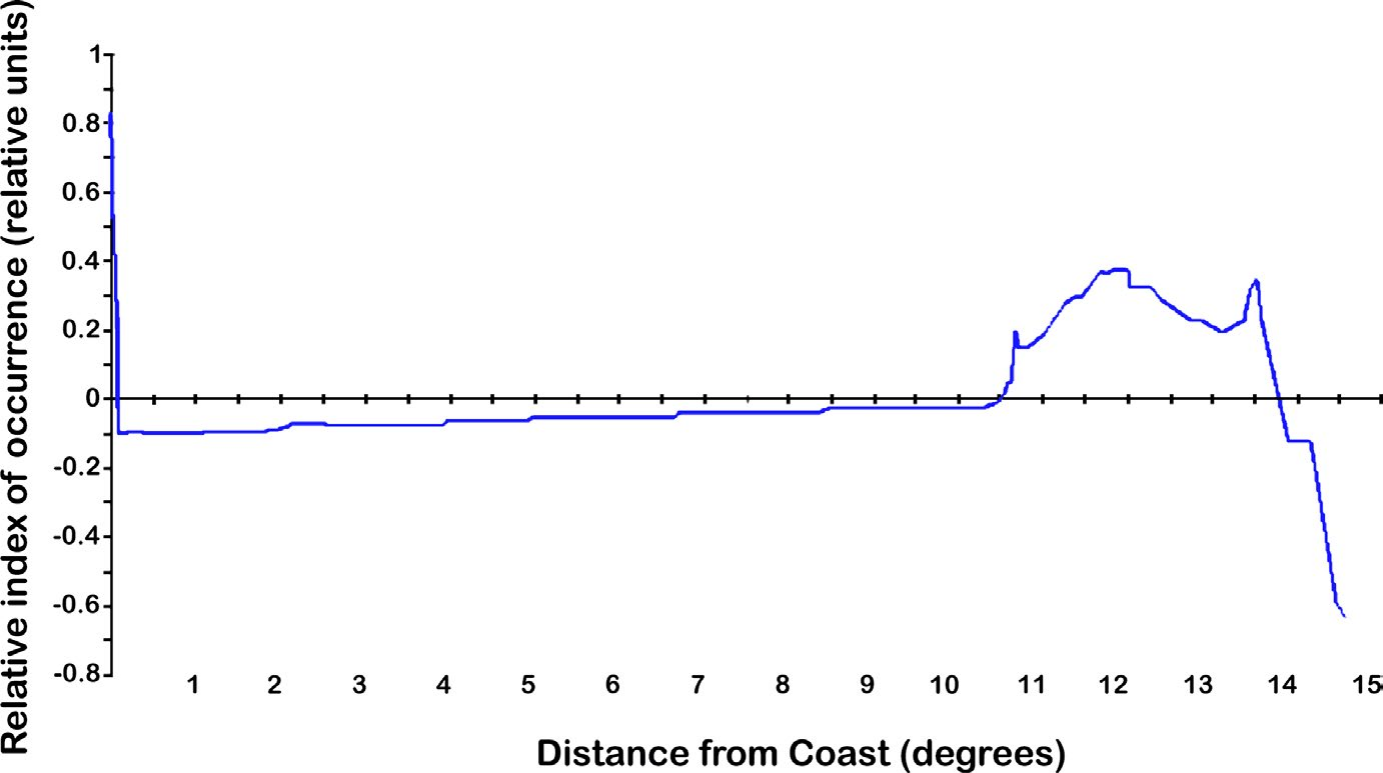
Relative index of occurrence as a function of distance to coast in the model output from model based on reported rabies cases in Canada. The data show that reported cases are distributed in a biphasic distribution in relation to distance to coast

The pattern of high relative predicted occurrence of reported rabies cases follows the distribution of actually reported cases but in addition the model predicts some areas of high relative occurrence in Prince Edward Island, southern coast of Hudson Bay and southern and extreme western NWT, which did not report cases or only single cases. Among environmental variables, human population density is the strongest predictor in our model (Figure 3), followed by distance to coast. Distance to coast showed a biphasic distribution with a peak close to coast and another about 11 –to 14 degrees distance (longitude and latitude) from coast (Figure 4).

**FIGURE 5:**
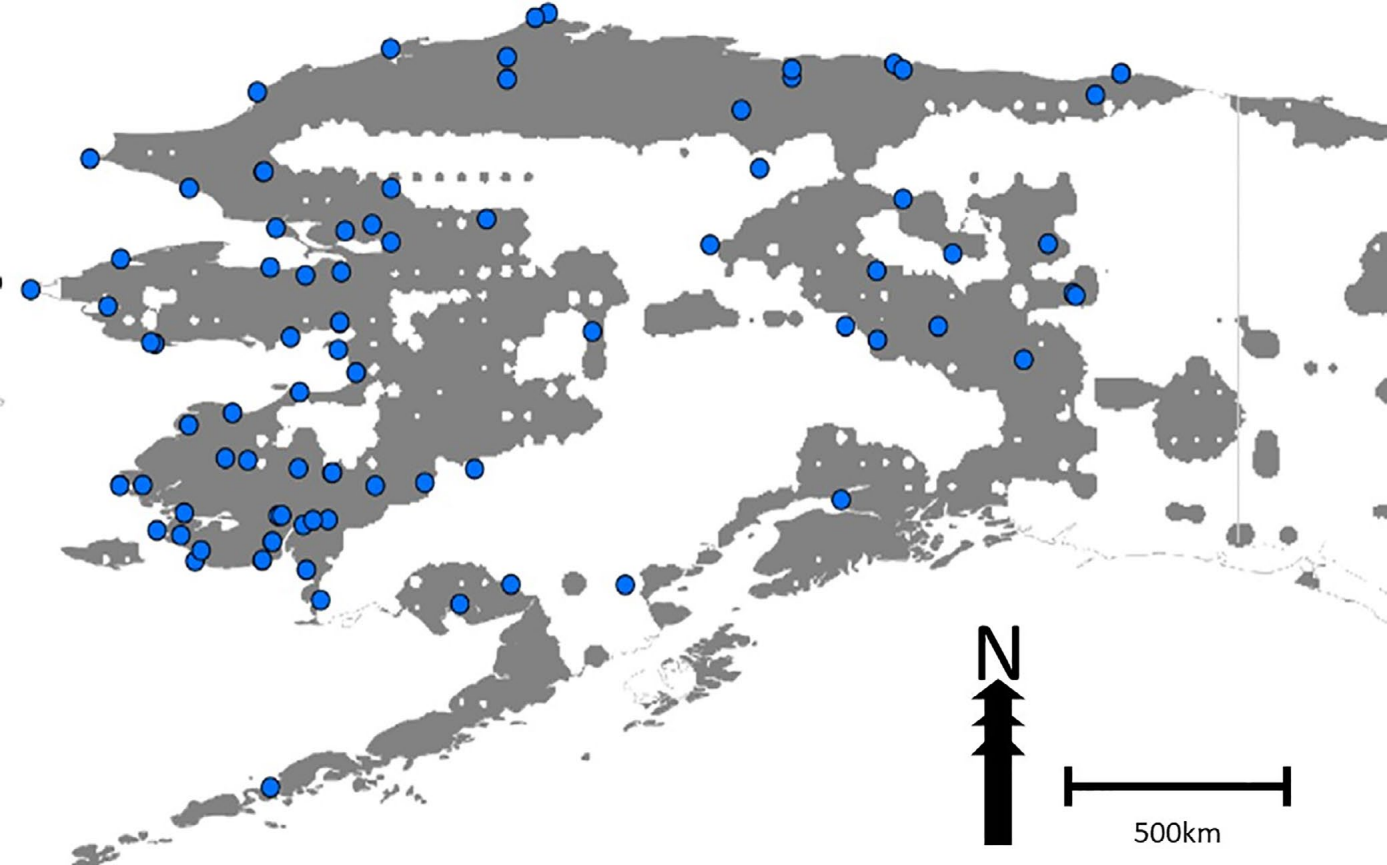
Overlay of ecological niche model, based on Canadian cases, onto Alaska. Grey area indicates +0.025 relative index of occurrence (RIO) from Figure 2 for binary display purposes. Blue dots indicate reported rabies cases (Huettmann et al., 2017)

To test how well the Canadian model generalizes and predicts reported rabies cases in adjacent Alaska, we compared the ecological niche model based on Canadian cases to Alaska (Huettmann et al., 2017) and found that the model trained on reported cases in Canada performs well in predicting reported rabies cases in Alaska. More than 95% of Alaskan reported rabies cases were successfully predicted by the model based on Canadian cases (Figure 5).

## 4 DISCUSSION

We report for the first time on a georeferenced nation‐wide Canadian rabies data set spanning the decade between 2007 and 2016 (Figure 1). We found that reported rabies cases in Canada are distributed unevenly in clusters with areas of regularly reported rabies infection and other areas with no reported cases in this time period. In an effort to better understand the ecological niche underlying this distribution, we employed a machine learning approach (Rojas et al., 2019) and 11 environmental predictors to develop an ecological niche model of relative index of occurrence (RIO) for reported rabies cases in Canada. Similar to our previous model for Alaska (Huettmann et al., 2017), the ecological niche model based on data from Canada predicts very well areas of presence and absence or rabies detection and reporting; human infrastructure was one of the major predictors of relative occurrence while distance to coast showed a biphasic relationship. This biphasic response likely represents the cases in the far north, east and west coasts versus those in Ontario and prairie provinces. The importance of closeness to human infrastructure, for example roads, industrial buildings and settlements, is likely driven by the nation‐wide sampling efforts that are associated with possible human exposure or public health measures that are also linked to human infrastructure. However, such biased sampling and reporting may not represent the only explanation. An influence of human activity on the ecology of rabies reservoir species might also contribute to this effect. Studies in Canada and the United States have demonstrated that raccoon population densities can be much higher in urban versus rural areas (Prange et al., 2003; Rosatte, 2000), and home ranges are smaller, strongly influenced by the availability of anthropogenic resources (Bozek et al., 2007). Similarly, arctic fox home ranges and diets have been shown to be influenced by human development sites such as petroleum development areas and air bases (Eberhardt et al., 1982; Kapel, 1999). To further understand the role of sampling bias or influence of human activity on underlying disease ecology in the maintenance hosts in this association with predicted occurrence of reported rabies cases, a more unbiased sampling regime is needed.

The ability of the model based on reported rabies cases in Canada to predict the reported rabies cases in Alaska suggests similar ecological drivers in determining the distribution of reported rabies cases throughout Northern North America. This is likely especially true for the cases in northern Canada, which showed close relationship to distance to coast, similar to endemic cases in Alaska. The similarity in ecological drivers could point to the possibility of a panarctic rabies management regime, at least in North America. In more southerly regions of the country, the model predicts areas of high RIO, for example, the provinces of Prince Edward Island and Nova Scotia, that have historically reported few cases. The predicted suitability of the habitat in these areas points to the importance of surveillance for imported cases.

One of the major limitations of this study is the human‐focused sampling effort. Given the current rabies sampling effort in Canada or Alaska, rabies occurrence cannot be resolved further for areas not affected by human access bias. Overall, our model is built on the best available data, which is heavily biased towards human exposure. Therefore, our ecological niche model can only confidently predict reported rabies cases. While reported cases clearly are related to underlying rabies ecology, to truly understand rabies dynamics in North America and its wilderness a more systematic unbiased surveillance approach is needed. Nevertheless, the model presented here provides functional model on the best available data under the current reporting protocols. At a minimum, it can serve as a starting point for enhanced surveillance efforts that could further inform public health and wildlife rabies management programming in Canada and Alaska. Such surveillance efforts may become of more interest as development in the North is predicted to expand in future years (Doods & Nutall, 2016). Similarly, parallel consideration of results from ecological niche and epidemiological (susceptible‐exposed‐infected‐removed) modelling approaches for arctic fox rabies could guide development of programs aimed at early detection of reintroduction of this virus variant into red fox populations of southern Quebec (Simon et al., 2019).

In this first study, cases from all regions of the country, and all species, regardless of the infecting virus variant, were combined. Future work will focus on identifying differences in the ecological niches of reported rabies cases in northern and southern Canada as well as parsing out cases due to the three predominant terrestrial virus variants (raccoon, western skunk and arctic) to further improve the performance of modelling efforts. Ideally, this model should be extended throughout northern America as well as Russia and other circumpolar regions.

## CONFLICTS OF INTEREST

The authors report no conflict of interest.

## Supporting information

Supplementary Material
